# Transmission of synovial sarcoma from a single multi-organ donor to three transplant recipients: case report

**DOI:** 10.1186/s13000-021-01181-5

**Published:** 2021-12-14

**Authors:** Jian Zhang, Yang Yang, Ye Tian, Ruifang Xu, Jun Lin

**Affiliations:** 1 Department of Urology, Beijing Friendship Hospital, Capital Medical University, 95 Yongan Road, Xicheng District, Beijing, China; 2 Beijing key laboratory of Tolerance Induction and Organ Protection in Transplantation, Beijing, 100050 China; 3 Department of Ultrasound, Beijing Friendship Hospital, Capital Medical University, Beijing, 100050 China

**Keywords:** Synovial sarcoma, Donor- transmitted malignancy, Donor transmission, Transplantation, Case report

## Abstract

**Background:**

Transmission of malignancy is a notable problem that cannot always be absolutely predicted at the time of transplantation. In particular, donor-derived transmission of synovial sarcoma in solid-organ transplantation is a rare but catastrophic event.

**Case presentation:**

We are the first to report three cases of synovial sarcoma transmitted from a single multi-organ donor in China. The donor died of respiratory failure caused by an intrathoracic tumor, which was diagnosed as benign at the time of donation. All three recipients developed synovial sarcoma 3–13 months after transplantation; all three cases were confirmed to be donor transmitted. The liver transplant recipient died of tumor metastasis after partial-allograft hepatectomy. The two renal-transplant recipients survived after comprehensive therapy, including allograft nephrectomy, withdrawal of immunosuppressants and targeted therapy with anlotinib.

**Conclusions:**

This report highlights the importance of detailed donor assessment, close follow-up and timely treatment of unexpected donor-transmitted malignancy. Although pathology is the most important evidence for the exclusion of donors for malignant potential, it should be combined with tumor type, tumor size and speed of growth. Organs from donors with malignant potential should be discarded. Allograft nephrectomy should be considered after confirmation of renal-allograft synovial sarcoma. Anlotinib for synovial sarcoma seems to be effective and well tolerated during long-term follow-up.

## Introduction

The persistent organ shortage requires maximum utilization of all available donors, including those with tumors, which can lead to donor-transmitted malignancy [1–3]. The consensus on whether to use donor organs with tumors is that it depends on the risk level of tumor transmission. However, malignancies can sometimes be misdiagnosed as benign at the time of donation, resulting in unexpected transmission. Herein, we report three cases of donor-transmitted synovial sarcoma from a single multi-organ donor in China. This is the first detailed case report of donor-derived synovial sarcoma transmission, including identification, diagnosis, clinical course, management and prognosis. This case will enhance our knowledge of tumor transmission in organ transplantation and help clinicians make well-informed assessments of donor risk. Additionally, our experience will also help optimize the diagnosis and treatment of donor-derived synovial sarcoma.

## Case presentation

### Transplant recipients

**Fig. 1:**
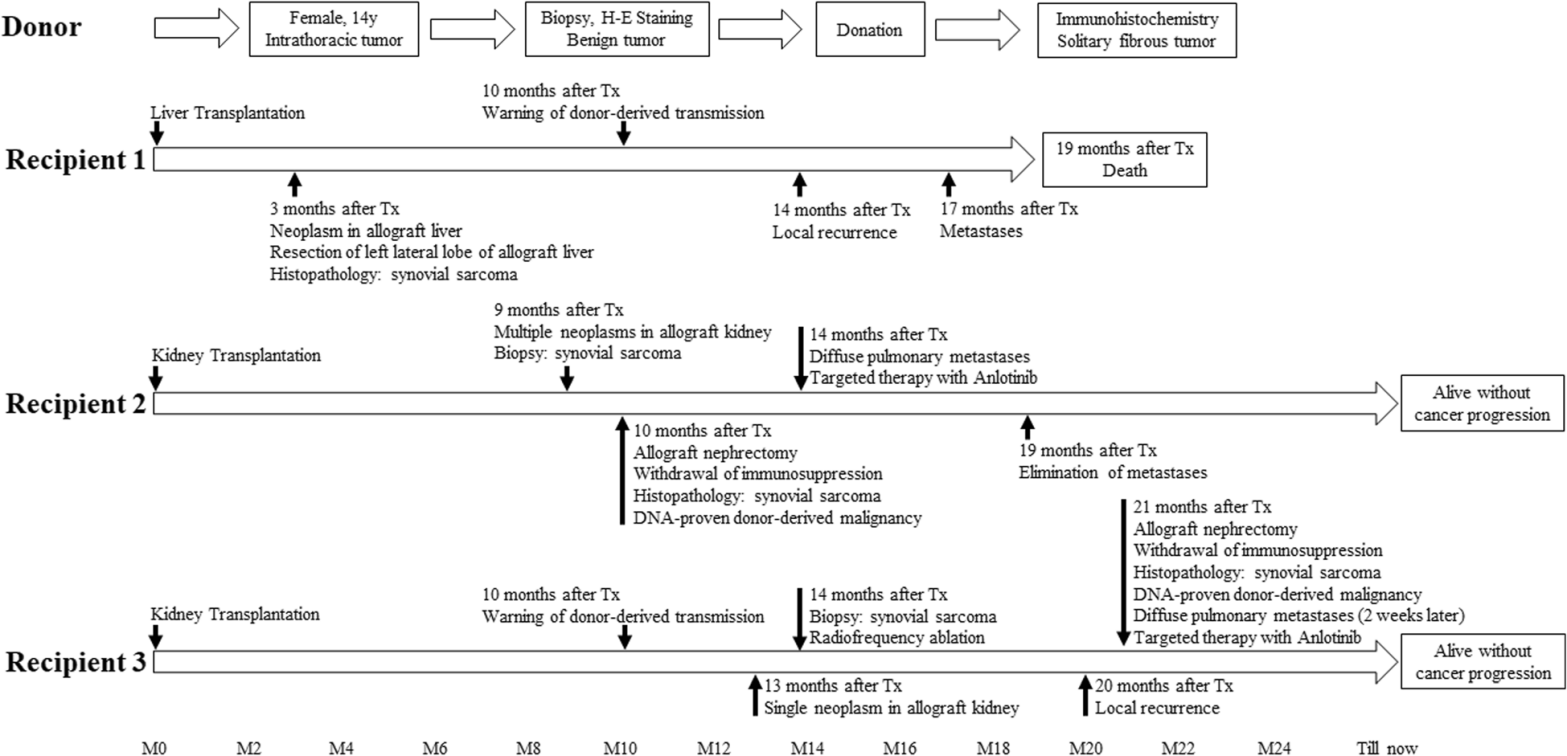
Clinical courses of three recipients and donor with donor-transmitted synovial sarcoma

Three patients who received organs from a single multi-organ donor consecutively developed allograft synovial sarcoma 3–13 months after organ transplantation. The timeline is shown in Fig. 1.

In recipient 1, synovial sarcoma initially developed in the allografted liver 3 months after transplantation. This patient was a 26-year-old female with primary liver cancer. However, donor transmission was not recognized. The patient underwent resection of the left lateral lobe of her allografted liver. Recurrence in the right lobe was found 11 months after partial-allograft hepatectomy. Three months later, systemic metastases were found, and the patient died within 2 months.

Recipient 2 was a 43-year-old male who received the donor’s left kidney. Nine months after transplantation, ultrasound (US) and computed-tomography (CT) scan found multiple neoplasms in the allografted kidney. Positron emission tomography with CT (PET/CT) revealed malignant tendency of neoplasms, but no distant metastasis. Synovial sarcoma was identified after a biopsy, and the patient received allograft nephrectomy followed by withdrawal of immunosuppression. Deoxyribonucleic acid (DNA) microsatellite ultimately proved the cancer to be donor transmitted. Four months after nephrectomy, CT examination revealed diffuse pulmonary metastases. The patient received long-term targeted therapy with anlotinib, which exhibited effective anti-tumor activity and eliminated metastases without any side effects. The patient survived without cancer progression over 2 years of follow-up.

Because his doctors had been warned of cancer transmission from the donor, recipient 3, a 33-year-old male who received the donor’s right kidney, underwent a regular cancer screening. Unfortunately, he developed a single neoplasm in the allografted kidney 3 months after the screening. Biopsy pathology revealed the same result in recipient 2. He initially received radiofrequency ablation (RFA) to preserve the allograft function. However, local recurrence was found 6 months later. Recipient 3 next underwent allograft nephrectomy, followed by withdrawal of immunosuppression. Donor transmission was proven by DNA microsatellite. Two weeks later, CT examination revealed diffuse pulmonary metastases, and the patient received long-term targeted therapy with anlotinib, which again eliminated pulmonary metastases without any side effects. This patient also survived without cancer progression over 2 years of follow-up.

### Organ donor

The donor was a 14-year-old female who developed a rapidly growing 11-cm intrathoracic tumor over the last 3 years of her life. Unfortunately, she died of respiratory failure caused by tracheal compression. Her liver and kidneys were donated after tumor diagnosis at the hospital of organ procurement: hematoxylin and eosin (H&E) staining of a biopsy performed before organ procurement seemed to indicate that the tumor was benign, and immunohistochemical (IHC) staining favored a diagnosis of solitary fibrous tumor (SFT). However, the subsequent occurrence of tumors in all three recipients and the pathological diagnoses of their allografts indicated that the pre-donation diagnosis was inaccurate.

### Radiographic findings

**Fig. 2:**
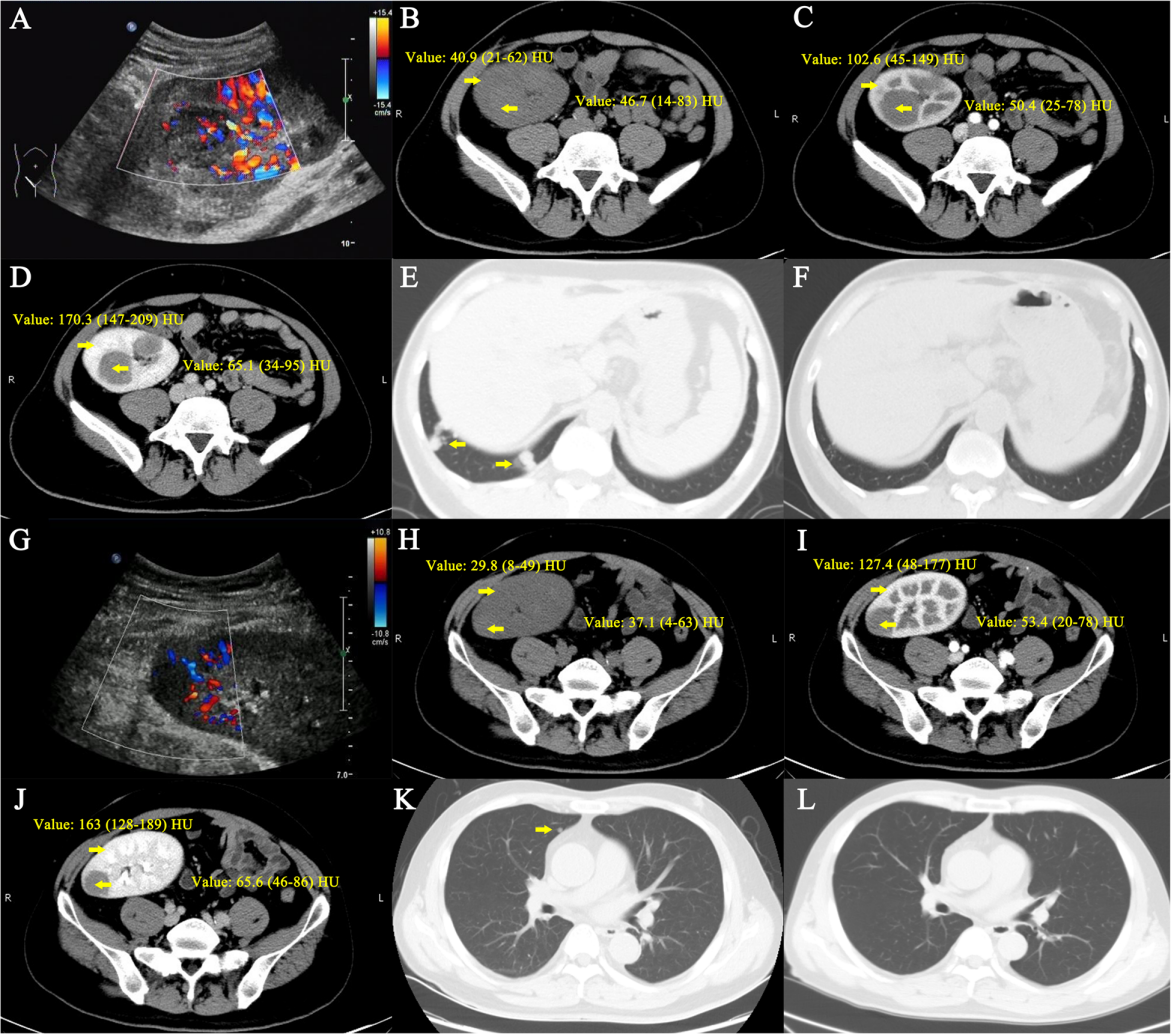
US and CT manifestations. Images **A–F** and **G–L** are from recipients 2 and 3, respectively. **A** and **G**: Color Doppler flow images revealed solid hypo-echo neoplasm with punctiform blood flow signals in the transplanted kidneys. **B** and **H**: Non-enhancing CT revealed slightly higher densities of neoplasms than allografts. **C**, **D**, **I**, **J**: Enhanced multiphase images revealed slow and relatively homogeneous enhancement inside the neoplasms, with lower densities than the allografts. **E** and **K**: CT revealed pulmonary metastases. **F** and **L**: CT revealed elimination of metastases after targeted therapy

**Fig. 3:**
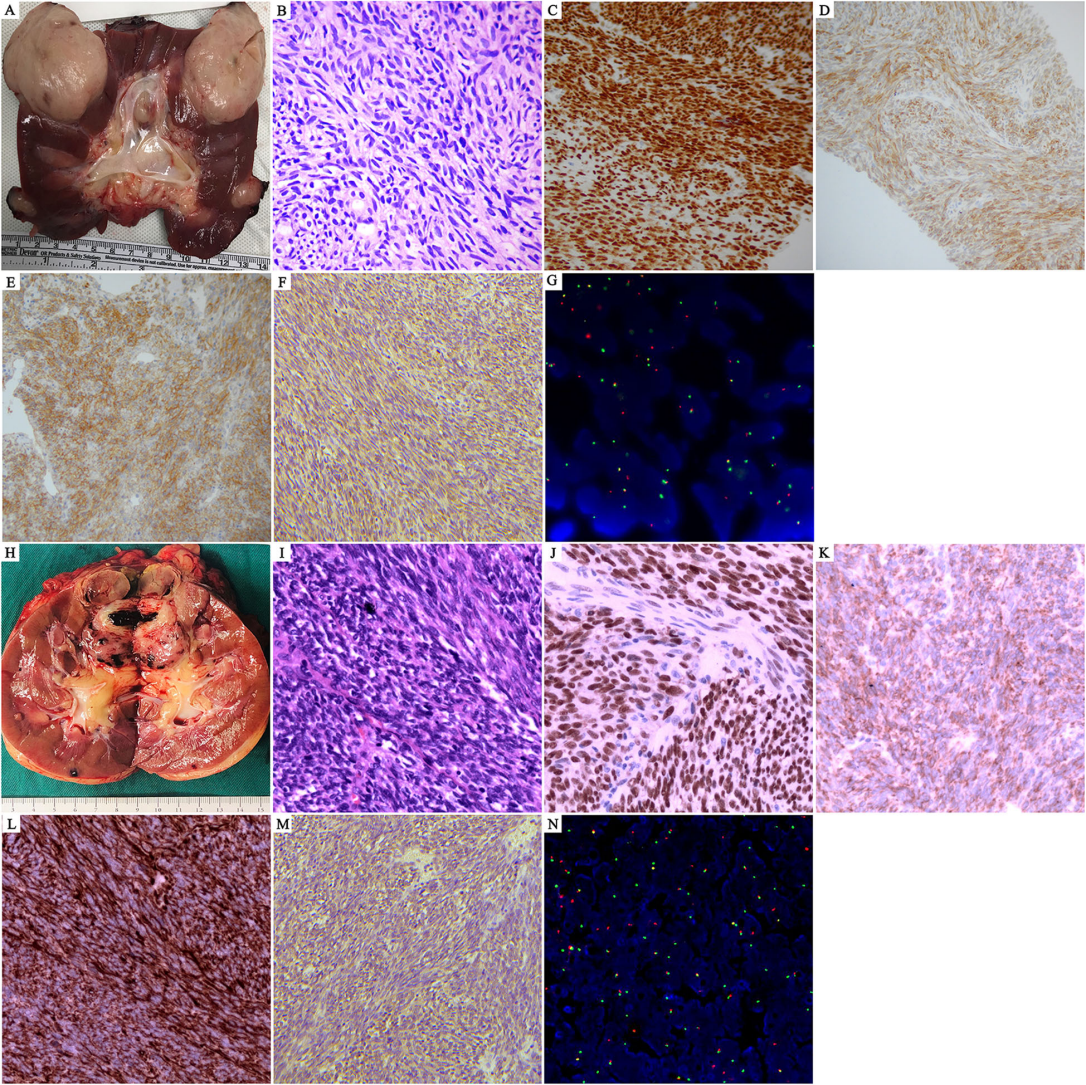
Histopathology of allograft synovial sarcoma. Recipient 2: **A**: specimen; **B**: H&E staining (200×); **C**: TLE1 (100×); **D**: Bcl-2 (100×); **E**: CD99 (200×); **F**: STAT6; **G**: FISH test; Recipient 3: **H**: specimen; **I**: H&E staining (200×); **J**: TLE1 (200×); **K**: Bcl-2 (200×); **L**: Vimentin (200×); **M**: STAT6; **N**: FISH test

Color Doppler US found multiple solid hypo-echo neoplasms with punctiform blood flow signal inside the allografted kidneys of recipients 2 (Fig. 2A) and 3 (Fig. 2G). Non-enhancing CT found slightly higher densities of neoplasms than those of the allografts, but slow and relatively homogeneous enhancement with lower densities inside the neoplasms appeared in enhanced multiphase images of recipients 2 (Fig. 2B–D) and 3 (Fig. 2H–J). Lung CT scan revealed elimination of pulmonary metastases after targeted therapy in recipients 2 (Fig. 2E–F) and 3 (Fig. 2K–L).

### Pathological findings

After allograft nephrectomy in recipients 2 and 3, the macroscopic cut surface showed a round sarcomatoid solid mass localized to the upper or lower pole of the transplanted kidney, with a tan-gray appearance and partial necrosis (Fig. 3A, H). The allografted tumors showed the histopathological characteristics of monophasic synovial sarcoma. H&E staining revealed entirely of infiltrative small hyperchromatic spindle cells with scant cytoplasm and indistinct cell borders, arranged in short, intersecting fascicles or in sheets (Fig. 3B, I). IHC staining showed that the tumor cells exhibited a strong and diffuse nuclear expression of transducin-like enhancer 1 (*TLE1*; Fig. 3C, J), a new nuclear marker for synovial sarcoma that is considered helpful in distinguishing synovial sarcoma from its histologic mimics, particularly when nuclear staining is moderate or strong [4]. The tumor cells also stained positive for B-cell lymphoma 2 (*Bcl-2*; Fig. 3D, K), cluster of differentiation 99 (*CD99*; Fig. 3E), signal transducer and activator of transcription 6 (*STAT6*) (Fig. 3F, M) and Vimentin (Fig. 3L) but were negative for cytokeratin (*CK*), CD34, integrase interactor 1 (*INI1*), desmin, myoblast determination 1 (*MyoD1*), Wilms tumor protein 1 (*WT-1*) and *S-100* protein (not shown). However, the expression of *STAT6* in tumor cells was mainly located in the cytoplasm rather than in the nucleus. Ultimately, molecular analysis confirmed the presence of synovial sarcoma translocation, chromosome 18 (SS18)–synovial sarcoma and X breakpoint 2 (SSX) fusion via fluorescent in situ hybridization (FISH) test, the gold standard in the diagnosis of synovial sarcoma, which demonstrated t(X;18)(p11.2;q11.2) translocation (Fig. 3G, N) [5].

### DNA microsatellite

**Fig. 4:**
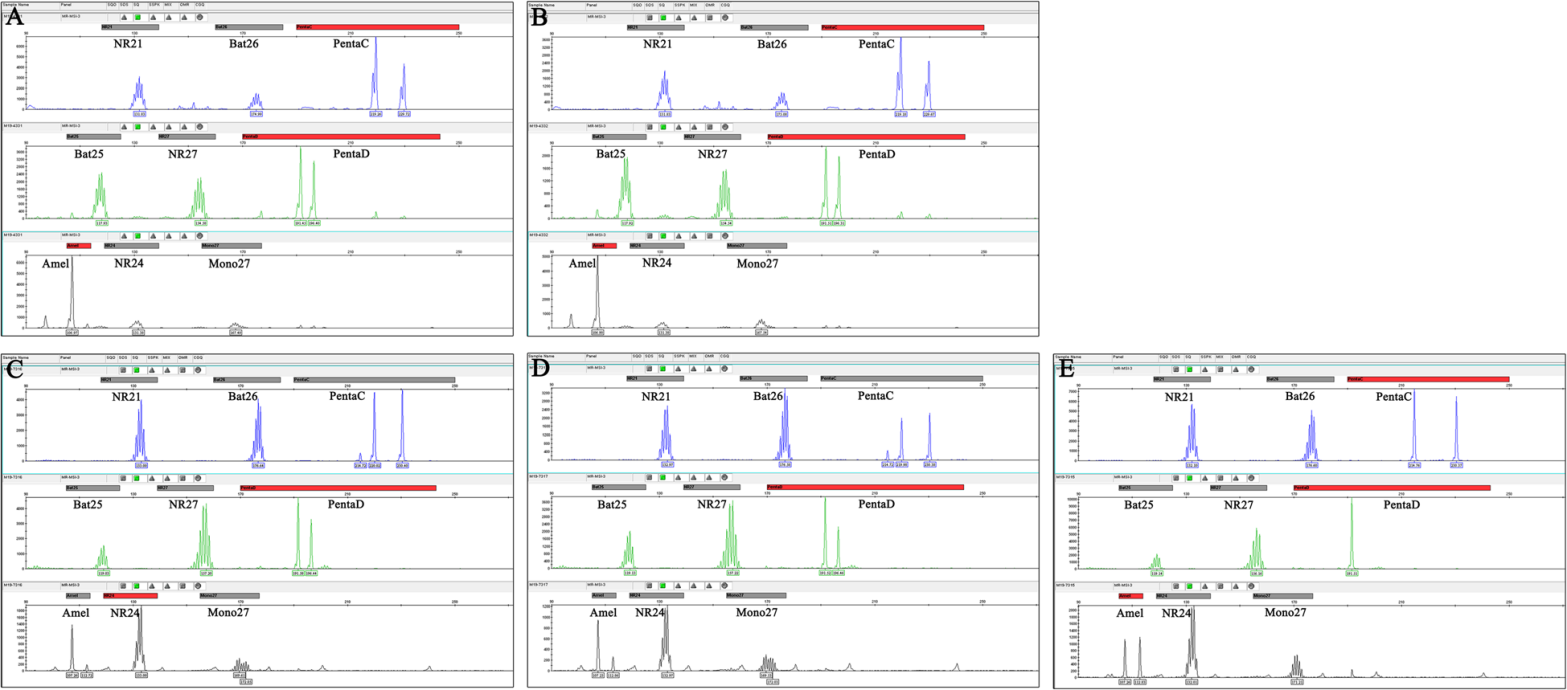
Length of detection locus as indicated by DNA microsatellite. Recipient 2: **A**: allograft tissue; **B**: tumor tissue. Recipient 3: **C**: allograft tissue; **D**: tumor tissue; **E**: blood of recipient. Nucleotide microsatellite loci: NR21, NR24, NR27, Bat25, Bat26, Mono27, PentaC, PentaD and Amel

DNA microsatellites are small-nucleotide repeats scattered throughout the genome. They are stably inherited and highly heterogeneous among individuals. Analysis of DNA microsatellites is, therefore, useful in transmitted malignancy to determine whether the tumor cells originated from the donor or the recipient [6]. The length of detection locus in different tissues is shown in Fig. 4. The figure indicates that the peripheral-blood nucleotide microsatellite loci of recipient 3 were completely different from those of the allograft and tumor tissues, while recipients 2 and 3 had identical loci between tumor tissues and the allografts. More importantly, the Amel locus exhibited female sex in both allograft and tumor tissues, confirming donor transmission.

## Discussion and conclusion

Malignancy derived from a donor organ is a rare event relative to the large number of transplants performed. However, once transmitted, it can often result in loss of graft function and/or high risk of mortality [7]. Recent transplantation registry reports estimate the risk of donor-derived tumor transmission to be 0.01–0.05% for each solid-organ transplant [7]. The most commonly transmitted cancer types are lymphoma (20.5%), renal cancer (17.9%), melanoma (17.1%) and lung cancer (10.3%). Melanoma and lung cancer have poor prognoses, with 5-year overall survival (OS) rates of 43 and 19%, respectively; meanwhile, renal cancer and lymphomas have favorable prognoses, with respective 5-year OS rates of 93 and 63% [8]. The incidence of sarcoma transmission is much lower; only a few cases of Kaposi’s sarcoma transmission have been reported [9]. Therefore, ours is the first report to describe synovial sarcoma transmission after a multi-organ procedure from a single donor to three recipients, including comprehensive diagnoses and treatment processes.

Transplantation from a donor with a benign tumor is safe and yields a valuable increase in organs for donation [10], but some benign tumors are difficult to distinguish from malignancies, which might increase the risk of tumor transmission. Identification of synovial sarcoma, which is mainly based on a combination of traditional morphology, identification of chromosomal t(X;18) translocation and a panel of IHC markers [11], remains a challenge due to its histological overlap with other soft tissue tumor types. In this case, synovial sarcoma was misdiagnosed as SFT at the time of donation, leading to the subsequent unexpected tumor transmission. Given that donor assessment and surgical organ procurement are often urgent, the evaluation of donor malignancies might not be comprehensive or may even be misdiagnosed. Because of its diverse histological features and involvement of diverse anatomical locations, SFT can mimic other soft tissue tumors of various lineages, including synovial sarcoma [4]. The IHC surrogate marker *STAT6* is proven to be a sensitive and specific molecular marker for genetic alteration (*NAB2-STAT6* gene fusion) in SFT, but *STAT6* can also be expressed in other soft tissue neoplasms, including synovial sarcoma [4]. As shown in this case, donor-derived synovial sarcoma can also express *STAT6* mainly in the cytoplasm. However, recent reports found that the expression of *STAT6* in SFT is exclusively nuclear, while other tumors may show both nuclear and cytoplasmic staining [4, 12]. Therefore, distinguishing donors’ soft tissue tumors from other tumors via pathological staining alone seems insufficient. Consulting an experienced pathologist and oncologist on the molecular pathological diagnosis and the biological behavior of the tumor should also be considered if the time for pre-donation assessment is sufficient. After reviewing the donor’s case, we found some evidence of malignant potential, including the large size and rapid growth of the tumor, which should have been taken into consideration along with pathological results. Previous studies have demonstrated a greater risk of metastases and poorer prognosis in either SFT or synovial sarcoma of a larger size [13, 14]. Therefore, micro-metastases might have occurred before the organ procurement procedure. Given the malignant potential in such an event, the organs should be discarded, especially if the diagnosis is unclear or controversial.

In this case, all recipients eventually developed synovial sarcoma, which indicated a high transmission rate. A previous study suggested that timely removal of the allograft might be beneficial to preventing the development of metastases [2]. In order to prevent transmission, this procedure should be considered in all recipients after notification of tumor transmission in one recipient from a multi-organ donor. If donor transmission had been recognized and warned for earlier, cancer in the recipients might have been prevented earlier by removal of the allografts.

Synovial sarcoma is considered aggressive and is noted for its propensity for local recurrence and metastasis, and its poor prognosis due to its radiation and chemotherapy resistance. Treatment of allograft synovial sarcoma is rarely reported. However, several therapeutic options for allograft renal mass exist, including partial nephrectomy, transplant nephrectomy, RFA and cryoablation, followed by altered or withdrawn immunosuppression [15]. Many transplant surgeons and urologists are faced with the dilemma of maximizing preservation of renal function while ensuring adequate cancer control. This report suggested that compared with local therapy, allograft nephrectomy followed by withdrawal of immunosuppression might be the best therapeutic option to prevent local recurrence, a finding that is supported by a previous study [3]. Nevertheless, it did not prevent the development of metastasis, probably due to the abovementioned delayed treatment. Anlotinib is a new oral tyrosine kinase inhibitor, primarily designed to inhibit multi-targets in vasculogenesis and angiogenesis, that exhibits direct anti-tumor activity in synovial sarcoma [16]. This is the first case of anlotinib being used in transplant recipients with synovial sarcoma; it proved to be effective, as indicated by the elimination of metastases and prolonged progression-free survival of the two renal-transplant recipients. It was also well tolerated during long-term follow-up. However, further observation of patients for drug resistance is needed.

This report highlights the importance of detailed donor assessment, close follow-up and timely treatment of unexpected donor-transmitted malignancy. Although pathology provides the most important evidence for the exclusion of donors due to malignant potential, it should be combined with tumor type, tumor size and speed of growth. Organs from donors with malignant potential should be discarded. Allograft nephrectomy should be considered after confirmation of renal-allograft synovial sarcoma. Anlotinib seems to be effective and well tolerated in synovial sarcoma patients during long-term follow-up.

### Patient perspective

Although it is regrettable that both renal-transplant recipients returned to dialysis after graft resection, they survived without further tumor spread due to aggressive and effective follow-up treatment. At present, both of them are satisfied with the overall treatment process and effect and show good compliance.

## Data Availability

All clinical data generated during this study are included in this article.

## Abbreviations

US: Ultrasound
CT: Computed-tomography
PET/CT: Positron emission tomography with CT
DNA: Deoxyribonucleic acid
RFA: Radiofrequency ablation
H&E: Hematoxylin and eosin
IHC: Immunohistochemical
SFT: Solitary fibrous tumor
TLE1: Transducin-like enhancer 1
Bcl-2: B-cell lymphoma 2
CD99: Cluster of differentiation 99
CK: Cytokeratin
INI1: Integrase interactor 1
MyoD1: Myoblast determination 1
WT-1: Wilms tumor protein 1
FISH: Fluorescent in situ hybridization
OS: Overall survival
STAT6: Signal transducer and activator of transcription 6
